# *Deinococcus radiodurans* Exopolysaccharide Inhibits *Staphylococcus aureus* Biofilm Formation

**DOI:** 10.3389/fmicb.2021.712086

**Published:** 2021-12-24

**Authors:** Fengjia Chen, Jing Zhang, Hyun Jung Ji, Min-Kyu Kim, Kyoung Whun Kim, Jong-Il Choi, Seung Hyun Han, Sangyong Lim, Ho Seong Seo, Ki Bum Ahn

**Affiliations:** ^1^Research Division for Radiation Science, Korea Atomic Energy Research Institute, Jeongeup, South Korea; ^2^Department of Biotechnology and Bioengineering, Chonnam National University, Gwangju, South Korea; ^3^Department of Oral Microbiology and Immunology, School of Dentistry, Dental Research Institute, Seoul National University, Seoul, South Korea; ^4^Department of Radiation Biotechnology and Applied Radioisotope Science, University of Science and Technology, Daejeon, South Korea

**Keywords:** exopolysaccharide, *Deinococcus radiodurans*, *Staphylococcus aureus*, biofilm formation, infection

## Abstract

*Deinococcus radiodurans* is an extremely resistant bacterium against extracellular stress owing to on its unique physiological functions and the structure of its cellular constituents. Interestingly, it has been reported that the pattern of alteration in *Deinococcus* proportion on the skin is negatively correlated with skin inflammatory diseases, whereas the proportion of *Staphylococcus aureus* was increased in patients with chronic skin inflammatory diseases. However, the biological mechanisms of deinococcal interactions with other skin commensal bacteria have not been studied. In this study, we hypothesized that deinococcal cellular constituents play a pivotal role in preventing *S. aureus* colonization by inhibiting biofilm formation. To prove this, we first isolated cellular constituents, such as exopolysaccharide (DeinoPol), cell wall (DeinoWall), and cell membrane (DeinoMem), from *D. radiodurans* and investigated their inhibitory effects on *S. aureus* colonization and biofilm formation *in vitro* and *in vivo*. Among them, only DeinoPol exhibited an anti-biofilm effect without affecting bacterial growth and inhibiting staphylococcal colonization and inflammation in a mouse skin infection model. Moreover, the inhibitory effect was impaired in the Δ*dra0033* strain, a mutant that cannot produce DeinoPol. Remarkably, DeinoPol not only interfered with *S. aureus* biofilm formation at early and late stages but also disrupted a preexisting biofilm by inhibiting the production of poly-*N*-acetylglucosamine (PNAG), a key molecule required for *S. aureus* biofilm formation. Taken together, the present study suggests that DeinoPol is a key molecule in the negative regulation of *S. aureus* biofilm formation by *D. radiodurans*. Therefore, DeinoPol could be applied to prevent and/or treat infections or inflammatory diseases associated with *S. aureus* biofilms.

## Introduction

*Deinococcus radiodurans* is a non-pathogenic bacterium extremely resistant to extracellular stresses, such as ionizing radiation, desiccation, UV radiation, and oxidizing agents (Fredrickson et al., 2008; Slade and Radman, 2011; Farci et al., 2016; Schmier et al., 2017). This exceptional resistance to multiple stresses results from the concerted actions of various physiological functions and well-regulated molecular mechanisms, including efficient DNA repair systems and enzymatic/non-enzymatic antioxidant systems (Makarova et al., 2001; Lim et al., 2019). In particular, *D. radiodurans* can protect against oxidative damage to important cellular components, such as proteins, nucleic acids, and lipids, *via* effective redox control and reactive oxygen species (ROS) scavenging (Chen et al., 2020). These features have been used in various industrial applications, such as decontamination of radioactive waste and development of cosmetic ingredients with the antioxidant and anti-aging functions (Xu et al., 2018; Lin et al., 2020).

*Deinococcus* has been reported as a commensal bacterium in various human tissues. Resident *D. radiodurans* has been found in healthy human skin (Chng et al., 2016). The Deinococcus-Thermus phylum has been reported in 23 gastric endoscopic biopsy samples and vaginal microbiota of healthy women (Bik et al., 2006; Diop et al., 2019). In addition, the beneficial role of *Deinococcus* in human skin has been speculated to be due to the quiet immune responses of host cells; however, its exact function has not been clarified (Ott et al., 2019). Interestingly, the proportion of *Deinococcus* is negatively correlated with skin inflammatory diseases, such as psoriasis or allergic skin inflammation, which may play a role in maintaining healthy skin (Yau et al., 2019; Guo et al., 2020). In contrast, other skin commensal bacteria, such as *Staphylococcus aureus, Corynebacterium*, and *Actinobacteria*, were detected at a high proportion in lesions in patients suffering from chronic skin inflammatory diseases, and it has been reported to be implicated in this etiology (Daly, 2009; Leyva-Castillo et al., 2020; Quan et al., 2020). However, the interactive function of *D. radiodurans* with other commensals and its effect on disease propensity has not been investigated.

*Staphylococcus aureus* is a Gram-positive opportunistic bacterium that commonly colonizes the skin, nose, and mucosal surfaces of healthy individuals (Schwartz et al., 2012; Truong-Bolduc et al., 2014; Mulcahy and McLoughlin, 2016). Approximately 20–30% of individuals are asymptomatically colonized by *S. aureus*, and 30% are intermittently colonized (Sakr et al., 2018). *S. aureus* is a leading cause of various infectious diseases, including pneumonia, sepsis, endocarditis, osteomyelitis, and skin and soft tissue infections (SSTIs). Skin tissue is the most common site for *S. aureus* colonization and infection, causing SSTI from minor, self-limiting, superficial infections to life-threatening diseases. *S. aureus*-associated SSTI may progress to invasive diseases, such as sepsis, endocarditis, and osteomyelitis (Olaniyi et al., 2017). *S. aureus* colonization and biofilm formation have been reported as universal behaviors that are significant risk factors for subsequent skin infections (Kwiecinski, 2015). When *S. aureus* forms a biofilm on the site of infection, it is 10–1,000 times more tolerant to antibiotics, antimicrobial peptides, and immune cell-mediated phagocytosis than the planktonic stage (Mah, 2012; Vestby et al., 2020).

There have been reports on the regulation of *S. aureus* biofilms *via* interactions with other commensal species. The negative regulatory effect of serine protease (ESP) from *Staphylococcus epidermidis*, a commensal bacterium, against *S. aureus* colonization has been shown in the nasal cavities of human volunteers (Iwase et al., 2010). We also previously reported that *S. aureus* biofilms were significantly inhibited by lipoteichoic acid derived from *Lactobacillus plantarum*, a probiotic commensal in the skin, gut, and oral cavity (Ahn et al., 2018). Understanding the interspecies interaction between commensals is important for understanding the pathology of inflammatory diseases or developing therapeutic strategies against these diseases. We hypothesized that *D. radiodurans* or its cellular constituents have beneficial effects on the host by playing a preventive role against *S. aureus* colonization and biofilm formation. Thus, we investigated the effect of *D. radiodurans* on *S. aureus* biofilm formation, including the underlying molecular mechanisms, and assessed its therapeutic potential to control *S. aureus* infection.

## Materials and Methods

### Bacteria and Reagents

The bacteria used in this study are listed in Supplementary Table 1. *D. radiodurans* strains were cultured at 30°C in tryptone glucose yeast extract (TGY) broth containing 0.5% tryptone, 0.3% yeast extract, and 0.1% glucose. *S. aureus* strains were cultured at 37°C in Luria-Bertani (LB) broth. All culture media used in this study were purchased from Difco (Franklin Lakes, NJ, United States). The LIVE/DEAD bacterial viability kit was purchased from Thermo Fisher Scientific (Waltham, MA, United States). The RNeasy Mini Kit was purchased from Qiagen GmbH (Hilden, Germany). The Primescript 1st strand cDNA synthesis kit was purchased from Takara Bio (Kusatsu, Japan). DNase I, RNase A, crystal violet solution, and Gram staining kit were purchased from Sigma-Aldrich (St. Louis, MO, United States). Wheat germ agglutinin conjugated with biotin (WGA-biotin) was purchased from GeneTex (Irvine, CA, United States). Horseradish peroxidase-conjugated streptavidin (streptavidin-HRP) was purchased from BD Biosciences (San Jose, CA, United States).

### Purification of DeinoPol

DeinoPols were prepared from *D. radiodurans* R1, KCTC13953BP, KCTC13954BP, or KCTC13955BP, as previously described (Lin et al., 2020). Briefly, *D. radiodurans* was cultured in TGY broth at 30°C for 48 h under shaking conditions. After incubation, 0.1% deoxycholate was added to the bacteria to lyse the cell wall, and the bacterial suspension was then heated at 100°C for 10 min to inactivate the enzymes. The supernatant was harvested by centrifugation (10,000 × *g*, 30min, 4°C), concentrated, and dialyzed using a minimal tangential flow filtration system with 30K Minimate capsules (Pall Life Sciences, Port Washington, NY, United States). The concentrate was precipitated with four volumes of 95% ethanol (Daejungchem, Seoul, Korea) at 4°C for 12 h to yield the crude polysaccharide. To remove proteins, the precipitate was suspended in distilled water and mixed with three volumes of chloroform:*n*-butanol (4:1v/v) for 20 min, and the aqueous phase was collected, followed by precipitation with 80% ethanol. Finally, it was filtered with a 0.22-μm Millex-GP syringe filter unit (Merck Millipore, Burlington, MA, United States) and lyophilized. In some experiments, DeinoPol was further treated with proteinase K (50 μg/ml) or DNase I (50 μg/ml) at 37°C for 1 h, or heat at 100°C for 10 min.

### Isolation of *Deinococcus radiodurans* Cell Wall Fraction (DeinoWall) and Cell Membrane Fraction (DeinoMem)

DeinoWall was isolated as previously described method (Wallinder and Neujahr, 1971), with some modifications. *D. radiodurans* was suspended in 1 M NaCl and disrupted using an ultrasonicator (Sanyo, Osaka, Japan), followed by removal of undisrupted cells or heavy cell debris from the bacterial lysates by centrifugation at 1,000 × *g* for 15 min. Next, the cytosolic proteins or light cell debris were removed by centrifugation at 18,800 × *g* for 15 min. The pellet containing DeinoWall was resuspended in 0.5% SDS in PBS and incubated at 60°C for 30 min to remove the cell membranous fraction. After centrifugation at 18,800 × *g* for 15 min, the pellets were resuspended in 1 M Tris-HCl and treated with 10 μg/ml DNase I and 50 μg/ml RNase A at 37°C for 2 h. After centrifugation at 18,800 × *g* for 15 min, the pellet was resuspended in 1 M Tris-HCl and incubated with 10 mM CaCl_2_ in the presence or absence of 200 μg/ml trypsin at 37°C for 18 h. After lyophilization, the quantity was determined by measuring the dry weight of DeinoWall and suspended in pyrogen-free water. To isolate DeinoMem, *D. radiodurans* was suspended in 0.1 M sodium citrate buffer (pH 4.7) and disrupted using an ultrasonicator. Bacterial lysates were vigorously mixed with an equal volume of *n*-butanol at RT for 30 min, and the aqueous phase was separated by centrifugation at 13,000 × *g* for 15 min. Butanol extraction was repeated three times. The aqueous phase was dialyzed against pyrogen-free water, followed by lyophilization and dissolution with pyrogen-free water.

### Preparation of Culture Supernatant (DRsup) and Heat-Killed Bacteria (HKDR) of *Deinococcus radiodurans*

To prepare DRsup, *D. radiodurans* was cultured in TGY broth at 30°C for 16 h under shaking condition, followed by dilution to prepare OD_600_ of 1 corresponds to 10^8^ CFU/ml. The culture was centrifuged at 10,410 × g for 10 min at 4°C. The culture supernatants were filtered through a 0.2 μm membrane filter to remove the remaining bacteria and debris, and then stored at −80°C. To prepare HKDR, the culture of *D. radiodurans* was centrifuged at 10,410 × g for 10 min at 4°C. After removal of the culture supernatant, the bacteria were washed and resuspended in PBS to a density of 10^9^ CFU/ml. The bacteria were heat-killed at 70°C for 30 min, and then stored in aliquots at −80°C. Complete killing was examined by plating on an TGY-agar plate overnight.

### Biofilm Assay With Crystal Violet Staining

*S. aureus* (5 × 10^7^ CFU/ml) was grown in 96-well plates (SPL, Pocheon, Korea) at 37°C for 24 h in LB broth in the presence or absence of DeinoPol. In some experiments, to investigate the biofilm prevention effect of DeinoPol, DeinoPol suspended in PBS was pre-incubated onto 96-well plates for 12 h at RT. *S. aureus* (5 × 10^7^ CFU/ml) was then grown in 96-well plates pre-coated with DeinoPol at 37°C for 24 h in LB broth. After incubation, planktonic bacteria were removed, and the biofilm was gently washed twice with PBS. The biofilms of *S. aureus* were stained with 0.1% crystal violet solution at RT for 30 min, followed by washing with PBS to remove non-specific stain. The adhered dye was dissolved in a solution (95% ethanol and 0.1% acetic acid), and absorbance was measured at 600 nm using a microplate reader (Biotec, Winooski, VT, United States).

### Confocal Laser Scanning Microscopy

*S. aureus* (5 × 10^7^ CFU/ml) was grown on sterile glass coverslips at 37°C for 24 h in LB broth in the presence or absence of DeinoPol. Planktonic bacteria were removed, and the biofilm was gently washed twice with PBS, followed by staining of the bacterial biofilm with SYTO9 and propidium iodide using the LIVE/DEAD bacterial viability kit according to the manufacturer’s instructions. After washing with PBS to remove non-specific stain, the biofilm was visualized using an LSM800 confocal laser scanning microscope (Zeiss, Jena, Germany). For quantification of the confocal microscopy data, 10 random independent fields of view per each group were selected and the mean fluorescence intensity (MFI) was analyzed using the ImageJ software.

### Quantitative Real-Time Reverse Transcription Chain Reaction

*S. aureus* (1 × 10^8^ CFU/ml) was grown on cell culture dishes (100 × 20 mm) for 12 h in LB broth in the presence or absence of DeinoPol (50 μg/ml). Bacteria were harvested, and total RNA was prepared using the RNeasy mini kit. Complementary DNA (cDNA) was synthesized from 5 μg of total RNA using the Primescript 1st strand cDNA synthesis kit. qRT-PCR was performed using SYBR Premix EX Taq (Takara Bio) in a real-time PCR system (Bio-Rad Laboratories, Hercules, CA, United States). The expression levels of these genes were normalized to the expression of *gyrB*. All primers were synthesized by Bionics (Seoul, Korea). The primer sequences are listed in Supplementary Table 2.

### Poly-*N*-Acetylglucosamine Detection

A crude PNAG extract was prepared as described previously (Toledo-Arana et al., 2005). Briefly, *S. aureus* (1 × 10^8^ CFU/ml) was grown in 1.7-ml microtubes at 37°C in LB broth in the presence or absence of DeinoPol, DeinoWall, or DeinoMem for 12 h. *S. aureus* was harvested by centrifugation at 10,000 × *g* for 5 min and washed five times with PBS. The pellets were then resuspended in 0.5 M EDTA (pH 8.0) and incubated at 100°C for 5 min. The supernatant was harvested by centrifugation at 10,000 × *g* for 10 min and treated with proteinase K (20 mg/ml) at 37°C for 30 min. The crude PNAG extracts were spotted onto a nitrocellulose membrane, and the blot was blocked with 5% skim milk in Tris-buffered saline with 0.1% Tween 20 (TBST) for 1 h. The membrane was then incubated overnight with 10 μg/ml WGA-biotin. After washing three times with TBST, PNAG was detected with streptavidin-HRP, followed by chemiluminescence detection using the ChemiDoc Touch Imaging System (Bio-Rad). The densities of PNAG were quantified using densitometry analysis in the ImageJ software.

### Biofilm Formation on HaCaT Cells Monolayer

HaCaT cells (5 × 10^4^ cells/well) were plated in a 96-well plate and grown until they reached confluence. *S. aureus* (5 × 10^5^CFU/well) was added to the cell monolayer in the presence or absence of 30 μg/ml of DeinoPol. The cells were then incubated at 37°C for 8 h in a humidified 5% CO_2_ incubator to form biofilms. After incubation, the biofilm was washed twice with PBS, and the cells were lysed with 0.1% Triton X-100. The number of the bacterial colonies was then counted.

### Staphylococcal Wound Infection Model

The animal experiments were approved by the Institutional Animal Care and Use Committee of the Korea Atomic Energy Research Institute (KAERI, IACUC-2019-03) and performed according to accepted veterinary standards by the KAERI Animal Care Center. Seven-week-old female BALB/c mice were obtained from Orient Bio (Seongnam, Korea). After 1 week of acclimatization, the mice were anesthetized by intraperitoneal administration of avertin (250 mg/kg), and a flat head of tack preheated in boiling water for 30 min was applied to the shaved dorsal of then mice for 30 s. *S. aureus* (1 × 10^7^ CFU) was then applied locally to the site of the burn wound in the presence or absence of DeinoPol. At 48 h post-infection, the mouse skin tissues were collected and subjected to Gram staining or homogenization for counting the bacterial CFU.

### Macrophage-Biofilm Interaction Assay

The experiment was performed as previously described (Ahn et al., 2018). Briefly, *S. aureus* (5 × 10^7^ CFU/ml) was incubated in LB broth on sterile glass coverslips at 37°C for 24 h in the presence or absence of DeinoPol. The biofilm was washed with PBS to remove LB broth and the planktonic bacteria. RAW264.7 murine macrophage cell line in FBS (fetal bovine serum) free-DMEM medium was added at 1 × 10^6^ cells/ml to the preformed biofilm and further incubated at 37°C for 2 h in a humidified incubator with 5% CO_2_, followed by washing with PBS to remove the left suspension bacteria and RAW 264.7 cells. Then, the remaining biofilm was detached, suspended in PBS, and plated onto LB agar plates. After 24 h of incubation, the number of bacterial colonies was determined.

### Statistical Analysis

The mean value ± standard deviation (S.D.) was obtained from triplicate samples for each treatment group. Statistical significance was determined by one-way ANOVA and Tukey post-test. Asterisks indicate significant induction compared with the control group (*P < 0.05, ^**^P < 0.01, and ^***^P < 0.001).

## Results

### *Deinococcus radioduran*s Inhibits *Staphylococcus aureus* Biofilm Formation

**FIGURE 1:**
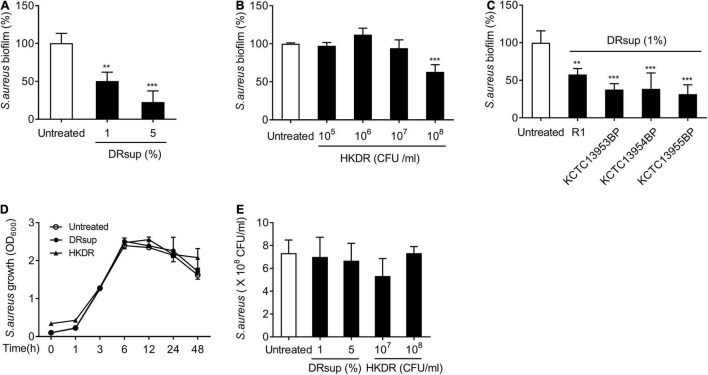
*D. radiodurans* inhibits *S. aureus* biofilm formation. **(A–C)** *S. aureus* (5 × 10^7^ CFU/ml) was grown on polystyrene plates at 37°C for 24 h in the presence or absence of **(A)** culture supernatants (DRsup) (1 or 5%) or **(B)** Heat-killed cells (HKDR) (10^5^, 10^6^, 10^7^, or 10^8^ CFU/ml) of *D. radiodurans* R1, or **(C)** DRsup of *D. radiodurans* KCTC13953BP, KCTC13954BP, or KCTC13955BP strains. Biofilms were determined by crystal violet assay. **(D)** *S. aureus* (1 × 10^7^ CFU/ml) was grown for 1, 3, 6, 12, 24, or 48 h in the presence or absence of HKDR (10^8^CFU/ml) or DRsup (5%) under shaking culture conditions. The growth of *S. aureus* was determined by optical density (O.D.) at 600 nm. **(E)** *S. aureus* (5 × 10^7^ CFU/ml) was grown at 37°C for 24 in the presence or absence of HKDR (10^7^ or 10^8^/ml) or DRsup (1 or 5%) under static culture conditions. Bacterial viability was measured by counting CFU. Data are the mean values ± S.D. of triplicate samples. Significant differences are indicated by asterisks (**P < 0.01 and ***P < 0.001).

To determine whether *D. radiodurans* can inhibit staphylococcal biofilm formation, we compared the biofilm formation of *S. aureus* in the presence of the indicated concentrations of deinococcal culture supernatant (DRsup) or heat-killed *D. radiodurans* R1 (HKDR). As shown in Figures 1A,B, DRsup potently inhibited biofilm formation by *S. aureus* in a dose-dependent manner and HKDR significantly inhibited biofilm formation at 10^8^ CFU/ml. To confirm, whether *Deinococcus* sp. inhibits staphylococcal biofilm formation, we tested three additional deinococcal strains, namely *D. radiodurans* KCTC13953BP, KCTC13954BP, and KCTC13955BP. All DRsup showed significant inhibitory effect on the biofilm production of *S. aureus* (Figure 1C). Next, we examined whether the anti-biofilm effect of *D. radiodurans* was due to its bactericidal effect on *S. aureus*. *S. aureus* was treated with DRsup or HKDR, and its growth and survival were measured. As shown in Figures 1D,E, neither DRsup nor HKDR altered *S. aureus* growth and viability. These data indicated that the inhibitory effect of *D. radiodurans* on staphylococcal biofilm formation was not due to a direct bactericidal effect, but may be caused by an indirect effect.

### DeinoPol Inhibits *Staphylococcus aureus* Biofilm Formation

**FIGURE 2:**
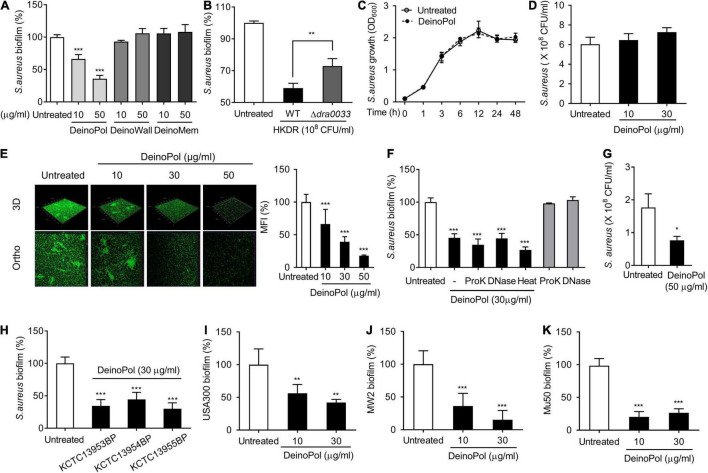
DeinoPol inhibits biofilm formation of *S. aureus*. **(A,B)** *S. aureus* (5 × 10^7^ CFU/ml) was grown on polystyrene plates at 37°C for 24 h in the presence or absence of **(A)** DeinoPol (10 and 50 μg/ml), DeinoWall (10 and 50 μg/ml), or DeinoMem (10 and 50 μg/ml) isolated from *D. radiodurans* R1, or **(B)** HKDR (1 × 10^8^ CFU/ml) of WT or Δ*dra0033* (DeinoPol-deficient strain) strains. Biofilms were determined by crystal violet assay. **(C)** *S. aureus* (1 × 10^7^ CFU/ml) was grown for 1, 3, 6, 12, 24, or 48 h in the presence or absence of DeinoPol (30 μg/ml) under shaking conditions. The growth of *S. aureus* was determined by O.D. at 600 nm. **(D)** *S. aureus* (5 × 10^7^ CFU/ml) was grown at 37°C for 24 h in the presence or absence of DeinoPol (10 or 30 μg/ml) under static culture condition. Bacterial viability was measured by counting CFU. Data are the mean values ± S.D. of triplicate samples. **(E)** *S. aureus* (5 × 10^7^ CFU/ml) was grown on coverslips at 37°C for 24 h in the presence or absence of DeinoPol (10, 30 or 50 μg/ml) and DeinoPol-treated *S. aureus* biofilms were visualized by confocal laser scanning microscopy (green from SYTO9 and red from propidium iodide). One of three similar results is shown. In the right panel, the MFI values of each group were quantified by ImageJ software. **(F)** *S. aureus* (5 × 10^7^ CFU/ml) was grown on polystyrene plates at 37°C for 24 h in the presence or absence of DeinoPol (30 μg/ml), 50 μg/ml of proteinase K (ProK)-treated DeinoPol (30 μg/ml), 50 μg/ml of DNase I (DNase)-treated DeinoPol (30 μg/ml), or heat-treated DeinoPol (30 μg/ml). Biofilms were determined by crystal violet assay. Data are the mean values ± S.D. of triplicate samples. **(G)** HaCaT cells monolayer was exposed to *S. aureus* (MOI = 10) at 37°C for 8 h in the presence or absence of 30 μg/ml DeinoPol. After removing the supernatant containing the planktonic bacteria, biofilms or bacteria adherent onto the cell monolayer were measured by counting CFU. **(H)** *S. aureus* (5 × 10^7^ CFU/ml) was grown on polystyrene plates at 37°C for 24 h in the presence or absence of DeinoPol (30 μg/ml) purified from *D. radiodurans* KCTC13953BP, KCTC13954BP, or KCTC13955BP strains. **(I)** *S. aureus* USA300, **(J)** MV2, or **(K)** Mu50 (5 × 10^7^ CFU/ml) were grown on polystyrene plates at 37°C for 24 h in the presence or absence of DeinoPol (10 or 30 μg/ml). Biofilms were determined by crystal violet assay. Data are the mean values ± S.D. of triplicate samples. Significant differences are indicated by asterisks (*P < 0.05, **P < 0.01, and ***P < 0.001).

Next, to identify the deinococcal component responsible for the inhibition of staphylococcal biofilm formation, major cell wall components were isolated, and their effects on biofilm formation were investigated. Three major representative cell wall components, namely exopolysaccharide (DeinoPol), cell wall (DeinoWall), and cell membrane (DeinoMem), were isolated and examined for their effects on *S. aureus* biofilm formation. DeinoPol markedly inhibited staphylococcal biofilm formation, whereas DeinoWall and DeinoMem did not (Figure 2A). As mentioned above, we found no direct bactericidal effect of DeinoPol on *S. aureus* (Figures 2C,D). To investigate whether DeinoPol is a major component of the cell wall fraction that is responsible for the inhibition of biofilm formation, we compared the biofilm inhibitory effect of WT and DeinoPol knockout mutants (Δ*dra0033*) (Lin et al., 2020). As shown in Figure 2B, the inhibitory effect of the WT was significantly weakened in Δ*dra0033*. Confocal microscopy analysis also showed that *S. aureus* biofilm formation was inhibited by DeinoPol in a dose-dependent manner; however, dead cells were rarely detected (Figure 2E). We previously demonstrated that DeinoPol is composed of 89.9% polysaccharides, 8.8% proteins and 1.3% DNA (Lin et al., 2020). To confirm polysaccharides are the major components of DeinoPol inhibiting biofilm formation, *S. aureus* biofilm formation was examined in the presence of proteinase K-treated DeinoPol, DNase I-treated DeinoPol, or heat-treated DeinoPol. As shown in Figure 2F, proteinase K, DNase I, and heat treatment did not significantly alter the inhibitory effects of DeinoPol on biofilm formation of *S. aureus*, indicating that the polysaccharides, the main component of DeinoPol, are predominantly responsible for the anti-biofilm effect of DeinoPol against *S. aureus*. Skin tissue is the most common site for *S. aureus* colonization and infection. To investigate whether DeinoPol interferes with staphylococcal colonization of skin cells, we used HaCaT cells, immortalized human keratinocytes, in a monolayer where *S. aureus* was grown to form biofilms in the presence or absence of DeinoPol. After 8 h of incubation, the biofilm bacteria were enumerated. As shown in Figure 2G, DeinoPol treatment considerably decreased biofilm bacterial burdens on HaCaT cell monolayers compared with the non-treatment group. Next, to examine whether the anti-biofilm effect of DeinoPol is a common characteristic of all *D. radiodurans*, DeinoPol was purified from various *D. radiodurans* strains, such as *D. radiodurans* KCTC13953BP, KCTC13954BP, and KCTC13955BP, and its effect on *S. aureus* biofilms was investigated. As expected, all tested DeinoPol significantly inhibited *S. aureus* biofilm formation at levels similar to those of DeinoPol isolated from the R1 strain (Figure 2H). Biofilm formation is considered an important mechanism in the pathogenesis of methicillin-resistant *S. aureus* (MRSA). Biofilms confer drug tolerance to broad-spectrum antibiotics, which contributes to the emergence of antibiotic-resistant bacteria, such as MRSA (Mirani and Jamil, 2011; Piechota et al., 2018). To confirm whether DeinoPol also inhibits the biofilm formation of MRSA, MRSA strains such as USA300, MW2, and Mu50 were treated with DeinoPol, and biofilm formation was examined. Figures 2I–K show that all tested MRSA strains showed significantly lower levels of biofilm formation after treatment with DeinoPol, indicating that DeinoPol might be a broad-spectrum inhibitor for various *S. aureus* strains including MRSA.

### DeinoPol Interferes With Biofilm Formation in the Early and Late Phases and Collapses Preexisting Biofilm

**FIGURE 3:**
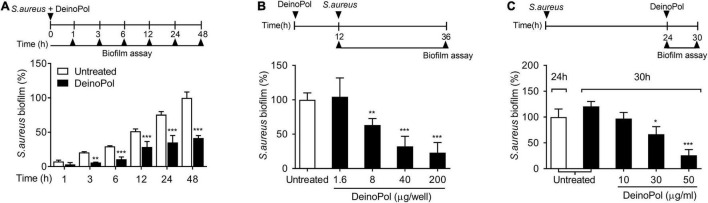
DeinoPol interferes with biofilm formation in the early and late phases and collapses preexisting biofilm. **(A)** *S. aureus* (5 × 10^7^ CFU/ml) was grown on polystyrene plates at 37°C for 1, 3, 6, 12, 24, or 48 h in the presence of DeinoPol (30 μg/ml). **(B)** *S. aureus* (5 × 10^7^ CFU/ml) was grown at 37°C for 24 h on polystyrene plates pre-coated with DeinoPol (1.6, 8, 40, or 200 μg/well) for 12 h. **(C)** Preexisting biofilm established with *S. aureus* (5 × 10^7^ CFU/ml) at 37°C for 24 h on polystyrene plates was treated with DeinoPol (10, 30, or 50 μg/ml) and further incubated for 6 h. Biofilms were determined by crystal violet assay. Data are the mean values ± S.D. of triplicate samples. Significant differences are indicated by asterisks (*P < 0.05, **P < 0.01, and ***P < 0.001).

To determine the phase of biofilm development at which the inhibitory effect of DeinoPol occurs, *S. aureus* biofilms formed for 1, 3, 6, 12, 24, or 48 h in the presence or absence of DeinoPol were measured. The inhibitory effect of DeinoPol was observed from 3 h after biofilm formation and lasted up to 48 h (Figure 3A). Next, we determined whether DeinoPol has preventive or destructive effects on biofilms. First, the plates were pre-coated with DeinoPol at various concentrations, and their effects on the biofilm-forming ability of *S. aureus* were investigated. Figure 3B shows that biofilm formation was hampered by DeinoPol at concentrations from 8 to 200 μg/well in a dose-dependent manner, but was not inhibited by DeinoPol at 16 μg/ml. To examine the destructive effect of DeinoPol against *S. aureus* biofilms, preexisting biofilms for 24 h were treated with DeinoPol for 6 h, and the biofilm was measured. *S. aureus* biofilm was significantly destroyed by DeinoPol at 30 and 50 μg/ml, but not at 10 μg/ml (Figure 3C). These results suggest that DeinoPol can inhibit *S. aureus* biofilm formation at the early and late stages with prophylactic and destructive effects.

### DeinoPol Regulates *ica* Gene Expression and Poly-*N*-Acetylglucosamine Production in *Staphylococcus aureus*

**FIGURE 4:**
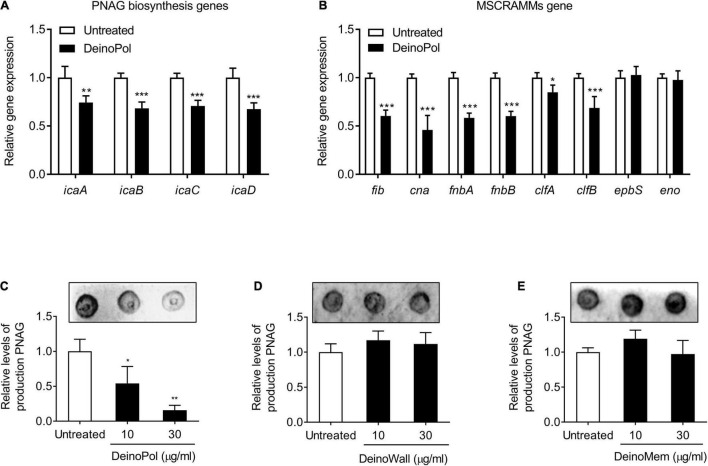
DeinoPol regulates *ica* gene expression and poly-*N*-acetylglucosamine production in *S. aureus*. **(A,B)** *S. aureus* (1 × 10^8^ CFU/ml) was treated with DeinoPol (50 μg/ml) for 12 h. Total RNA was isolated, and the mRNA expression levels of **(A)** *ica* genes (*icaA*, *icaB*, *icaC*, and *icaD*) and **(B)** microbial surface components recognizing adhesive matrix molecules (MSCRAMMs) genes (*fib*, *cna*, *fnbA*, *fnbB*, *clfA*, *clfB*, *ebpS*, and *eno*) were examined by qRT-PCR analysis. The expression of *gyrB* was used for q-PCR normalization. Data are the mean values ± S.D. of triplicate samples. **(C–E)** *S. aureus* (1 × 10^8^ CFU/ml) was treated with DeinoPol (10 or 30 μg/ml), DeinoWall (10 or 30 μg/ml), or DeinoMem (10 or 30 μg/ml) for 12 h. The crude extract of PNAG from *S. aureus* was blotted on nitrocellulose membrane and detected by WGA-biotin/HRP-streptavidin, followed by visualization with chemiluminescence detection. One of three similar results is shown. In the lower panel, the densities of PNAG were quantified by densitometry analysis. Data are the mean values ± S.D. of triplicate samples. Significant differences are indicated by asterisks (*P < 0.05, **P < 0.01, and ***P < 0.001).

The intracellular adhesion (*ica*) locus is responsible for the production of poly-*N*-acetylglucosamine (PNAG), an essential component for staphylococcal biofilm formation (O’Gara, 2007). In addition, microbial surface components recognizing adhesive matrix molecules (MSCRAMMs) genes, such as *fib* (fibrinogen binding protein), *cna* (collagen binding protein), *fnbA* (fibronectin binding protein A), *fnbB* (fibronectin binding protein B), *clfA* (clumping factor A), *clfB* (clumping factor B), *ebps* (elastin-binding protein), and *eno* (laminin-binding protein), have been reported to be involved in the attachment of staphylococci. To identify the underlying mechanisms for biofilm inhibition by DeinoPol, the regulatory effect of DeinoPol on the expression of these staphylococcal biofilm-associated genes was examined. As shown in Figure 4A, when *S. aureus* was incubated with DeinoPol (50 μg/ml), the mRNA expression of icaABCD was significantly downregulated. Moreover, the mRNA expression of *fib*, *cna*, *fnbA*, *fnbB*, *clfA*, and *clfB* was suppressed by DeinoPol treatment, but *ebps* and *eno* expression was not affected (Figure 4B). Next, the production of PNAG was measured in *S. aureus* treated with DeinoPol, DeinoWall, or DeinoMem. Figures 4C–E show that PNAG production was inhibited by DeinoPol in a dose-dependent manner, whereas DeinoWall and DeinoMem did not affect to PNAG. These results suggest that DenoPol inhibits *ica* gene expression and PNAG production, contributing to the inhibition of *S. aureus* biofilm formation.

### DeinoPol Inhibits *Staphylococcus aureus* Burdens in Skin Wound Infection Model

**FIGURE 5:**
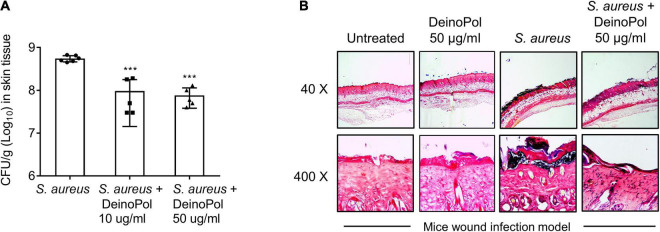
DeinoPol inhibits *S. aureus* burdens in skin wound infection model. **(A,B)** *S. aureus* (1 × 10^7^CFU) was inoculated to the dorsal burn wound of mice in the presence or absence of DeinoPol (10 or 50 μg/wound) for 48 h to allow biofilm formation. **(A)** *S. aureus* infected-skin tissues were homogenized by using a 2-mm homogenizer bead, and bioburden was calculated by counting CFU. Six and five mice were used in the non-treatment and DeinoPol-treatment groups, respectively. Asterisks indicate significant induction compared with the non-treatment group (***P < 0.001). **(B)** The tissue sections were subjected to Gram-staining and imaged at 40 × or 400 × magnification. One of five similar results is shown.

The inhibition of *S. aureus* biofilm formation by DeinoPol *in vivo* was evaluated in a mouse model of burn wound biofilm. *S. aureus* was treated to the mouse dorsal burn wound in the presence or absence of DeinoPol for 48 h to allow biofilm formation and the amount of biofilm bacteria was calculated by counting colony-forming units. Figure 5A shows that the average bioburden on the mouse wound skin surface was 5.5 × 10^8^ CFU/g tissue in the untreated group. However, this average of bioburden was decreased to 9.6 × 10^7^ CFU/g and 7.6 × 10^7^ CFU/g by treatment with 10 and 50 μg DeinoPol, respectively. Gram staining analysis of the skin tissue also showed that a dense bacterial community (deep violet) was observed below the epidermis of the skin in the *S. aureus* infection group, and it was substantially reduced in the DeinoPol treatment group (Figure 5B). These results suggest that DeinoPol exerts anti-biofilm effects *in vivo*, implying its potential use as a clinical treatment for infectious diseases associated with *S. aureus* biofilms.

### DeinoPol Enhances the Antibacterial Susceptibility of *Staphylococcus aureus* in Biofilms

**FIGURE 6:**
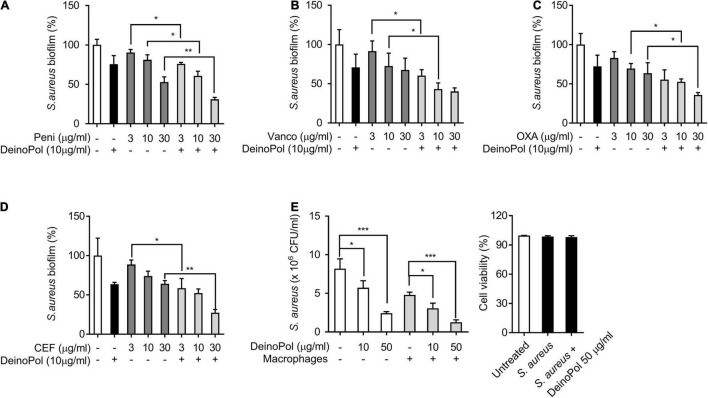
DeinoPol enhances the antibacterial susceptibility of *S. aureus* in biofilm. **(A–D)** *S. aureus* (5 × 10^7^CFU/ml) was grown on polystyrene plates with DeinoPol (10 μg/ml) at 37°C for 24 h, and further incubated for 6 h in the presence or absence of **(A)** penicillin (3, 10, or 30 μg/ml), **(B)** vancomycin (3, 10, or 30 μg/ml), **(C)** oxacillin (3, 10, or 30 μg/ml), or **(D)** cefazolin (3, 10, or 30 μg/ml). Biofilms were determined by crystal violet assay. **(E)** *S. aureus* (5 × 10^7^CFU/ml) was grown on coverslips at 37°C for 24 h with DeinoPol (10 or 50 μg/ml) and RAW 264.7 cells (1 × 10^6^ cells/ml) were added to the preformed biofilm and further incubated at 37°C for 2 h. The remaining biofilms were measured by counting colony forming units (left panel) and the cell viability was assessed by flow cytometry after staining with propidium iodide (right panel). Data are the mean values ± S.D. of triplicate samples. Significant differences are indicated by asterisks (*P < 0.05, **P < 0.01, and ***P < 0.001).

Biofilm resistance to antibiotics has been considered a public health concern owing to the improper use and overuse of antibiotics (Sharma et al., 2019). To examine if DeinoPol increases the inhibitory effect of antibiotics on *S. aureus* biofilm formation, *S. aureus* biofilm was treated with various antibiotics clinically used for *S. aureus* infection, such as penicillin, vancomycin, oxacillin, and cefazolin, in the presence or absence of DeinoPol, and then the biofilm was measured. As shown in Figures 6A–D, DeinoPol enhanced the capacity of all tested antibiotics to inhibit *S. aureus* biofilms. Although macrophages are major innate immune defenders against microbial infection, *S. aureus* biofilms have been reported to prevent macrophage phagocytosis and avoid immune responses (Thurlow et al., 2011). The effect of DeinoPol on macrophage function to eradicate *S. aureus* biofilms was examined. Pre-incubated *S. aureus* biofilm with DeinoPol was treated with RAW264.7 cells, and the degree of biofilm reduction and the cell viability were measured. Figure 6E shows that DeinoPol enhanced the removal effect of macrophages against *S. aureus* biofilm (left panel). The macrophage viability was not altered (right panel). These results indicate that DeinoPol can improve the susceptibility of *S. aureus* biofilms to antibiotics and macrophages.

## Discussion

*D. radiodurans*, an extremophile bacterium, is a normal inhabitant flora of the human body, but its interactive role in the defense against pathogen infection has not been studied. In this study, we revealed that *D. radiodurans* inhibited *S. aureus* biofilm formation. Specifically, DeinoPol acted as a major component of *D. radiodurans*, inhibiting biofilm formation without affecting bacterial growth by abrogating the expression of the *ica* genes required for PNAG production. Furthermore, DeinoPol enhanced the susceptibility of biofilms to antibiotics and macrophages. These results indicate that *D. radiodurans* functions as a commensal that prevents *S. aureus* infection, and provide a compelling evidence that DeinoPol can be a potential antimicrobial agent for controlling *S. aureus* biofilm formation.

DeinoPol, not DeinoWall and DeinoMem, was identified as a functional constituent of *D. radiodurans* to inhibit biofilm formation by *S. aureus* in this study. Moreover, this inhibitory function was not confined to DeinoPol of the type strain, but was also possessed by the DeinoPol of *D. radiodurans* KCTC13953BP, KCTC13954BP, and KCTC13955BP strains isolated from Baekrokdam, Jeju, Republic of Korea. These results are consistent with previous reports showing the inhibitory function of extracellular galactan from *Kingella kingae* against biofilm formation by *S. aureus*, *S. epidermidis*, *Candida albicans*, and *Aggregatibacter actinomycetemcomitans* (Bendaoud et al., 2011), and inhibition of enterohemorrhagic *Escherichia coli* biofilm formation by the EPS released by *Lactobacillus acidophilus* (Kim et al., 2009). In contrast, EPS composes up to 40% of the dry weight of dental plaque, and glucan is essential for biofilm formation by *Streptococcus mutans* (Koo et al., 2010). An EPS-deficient strain of *Bacillus subtilis*, Δ*eps*H, formed smooth colonies and fragile pellicles compared with the WT strain (Epstein et al., 2011). This contradictory effect of EPS on bacterial biofilm formation is due to the diverse structures and compositions of each bacterial species (Joseph and Wright, 2004; Jiang et al., 2011). We previously showed that DeinoPol is composed of xylose, galactose, fucose, glucose, arabinose, and fructose in a molar ratio of 10.6:6.1:4.2:3.8:2.6:1.0, and 14.89% of unknown sugars (Lin et al., 2020). This oligosaccharide composition is similar to that of *Lactobacillus plantarum* WLPL04 EPS, which consists of xylose, glucose, and galactose at a molar ratio of 3.4:1.8:1 and exerts an anti-biofilm activity against *Pseudomonas aeruginosa* CMCC10104, *Escherichia coli* O157:H7, *Salmonella typhimurium* ATCC13311, and *S. aureus* CMCC26003 (Liu et al., 2017). In particular, DeinoPol contains fucose, which is considered a rare sugar contained in bacterial EPS and has been reported to have an inhibitory function on the biofilm of formation several bacteria (Khodse and Bhosle, 2010). L-Fucose inhibited the biofilm formation of *Campylobacter jejuni* NCTC11168, but not that of the fucose permease mutant strain (Dwivedi et al., 2016). L-Fucose and trithiotriazine-cored L-fucose cluster inhibited biofilm formation by *P. aeruginosa* through binding with LecB, a lectin of *P. aeruginosa*, and this effect was also observed in *P. aeruginosa*-infected cystic fibrosis patients (Hauber et al., 2008; Smadhi et al., 2014; Grishin et al., 2015). These previous reports support that the unique oligosaccharide composition and fucose content may play a crucial role in the anti-biofilm activities of DeinoPol.

Here, we showed that DeinoPol inhibited the biofilm formation of *S. aureus* in the early and late phases, and even destroyed preformed biofilms. This implies that the inhibition of biofilm formation by DeinoPol might be elicited by several distinct mechanisms. Carbohydrate, the major component of EPS, confers an anionic property to the bacterial EPS, which has been reported to interfere with the prerequisite for biofilm formation, such as cell-surface and cell-cell interactions, by electrostatic modifications (Valle et al., 2006; Jiang et al., 2011). The ability of DeinoPol to destroy the preformed biofilms is coincident with previous resports, such as dispersion of *P. aeruginosa* biofilm by A101 polysaccharide purified from *Vibrio* sp. QY101 (Jiang et al., 2011) and disruption of preformed biofilm of *S. aureus* and *S. epidermidies* by *Kingella kingae* exopolysaccharide (Bendaoud et al., 2011). However, the precise mechanisms by which the preformed biofilms are destroyed by bacterial polysaccharide are still unknown. It has been reported that bacterial polysaccharides act as a signaling molecule that can regulate gene expression of bacteria (Kim et al., 2009), which encourages speculation that DeinoPol induces the production of self-produced factors of *S. aureus* mediating biofilm disassembly, such as phenol soluble modulins (PSMs) or extracellular proteases (Boles and Horswill, 2008; Otto, 2013), *via* regulating gene expression in *S. aureus*. Further studies are required to elucidate the exact mechanisms of the potential receptor for DeinoPol and its signaling pathway modulating the expression of molecules known to degrade PNAG and *S. aureus* biofilm.

*S. aureus* in biofilms is 10–1,000 times more resistant to antibiotics and phagocytosis by macrophages and neutrophils than the planktonic bacteria, and this is associated with chronic inflammation (Mah, 2012). Hence, there have been recent attempts to use additives to enhance the susceptibility of bacterial biofilms to antibiotics or immune cells (Verma et al., 2009; Ferrer-Espada et al., 2020). As we found in this study, DeinoPol increased the anti-biofilm effect of antibiotics and macrophages. It is hypothesized that the biofilm matrix polymer is loosened by DeinoPol by regulating PNAG production, which eases the penetration of antibiotics. Inhibition of PNAG production by DeinoPol might be a key mechanism for enhancing the susceptibility of *S. aureus* biofilms to antibiotics, as PNAG has been reported to act as a physical barrier for *S. aureus* biofilms, conferring resistance to antibiotics and the immune system (Brooks and Jefferson, 2014). Molecules that can dissemble bacterial biofilms by degrading or regulating EPS synthesis are referred to as biofilm-dispersing agents, and they can be applied to various bacterial biofilms detected in the environment or clinics (Verderosa et al., 2019). For example, chitosan, D-amino acids, phenol-soluble modulins, and dispersin B are dispersing agents that enhance the susceptibility of biofilms to antibiotics by inducing the release of planktonic cells from biofilms (Alksne and Projan, 2000; Izano et al., 2007; Mu et al., 2014; Sanchez et al., 2014). This study confirms that DeinoPol is a potential biofilm-dispersing agent against *S. aureus*.

DeinoPol regulated the biofilm formation of *S. aureus* laboratory strains as well as MRSA isolates, suggesting that DeinoPol could be widely used to inhibit the biofilm formation of various *S. aureus* strains. Most natural biofilms are composed of multispecies bacteria (Yang et al., 2011). In particular, *S. aureus* has been reported to form a multispecies biofilm with *P. aeruginosa*, *Salmonella* spp., *Enterococcus faecalis*, *Cutibacterium acnes*, *C. albicans*, and *S. epidermidis* in various tissues (Citron et al., 2007; Pathak et al., 2012; Gannesen et al., 2018; Iniguez-Moreno et al., 2018; Trizna et al., 2020). Multispecies biofilms strengthen their protective effect against hostile environments *via* cell-cell communication by quorum sensing or diffusible signals, genetic exchanges, or physical interaction (Li and Tian, 2012; Rendueles and Ghigo, 2012; Hansen et al., 2017; Krzyzek and Gosciniak, 2018). Therefore, it is important to clarify the anti-biofilm effect of candidate molecules against single-species biofilms as well as multispecies biofilms. The regulatory effect of DeinoPol against multispecies biofilms containing *S. aureus* was not determined in this study, but should be investigated in future studies to advance the application of DeinoPol as a clinical treatment for *S. aureus* infectious diseases.

Although Δ*dra0033* strain, DeinoPol-deficient strain, was treated to *S. aureus* biofilm, approximately 30% *S. aureus* biofilm was reduced. This implies that unknown substances other than DeinoPol may be slightly involved in the residual effect of Δ*dra0033* strain on biofilm inhibition. Various substances derived from microorganism have been reported to inhibit *S. aureus* biofilm formation. For example, autoinducer-2 (AI-2), a quorum-sensing signaling molecule, is known to inhibit biofilm of *S. aureus* by down-regulating the transcription of *icaA* (Yu et al., 2012). AI-2 has also been reported as a major quorum-sensing molecule to regulate physiological functions in *D. radiodurans* (Lin et al., 2016), which may have acted as a substance responsible for residual inhibitory effect of Δ*dra0033* strain. *D. radiodurans* highly expresses carotenoids that play an important role in the antioxidant effects of the strain through resistance to ROS. Carotenoids have been reported to exhibit the anti-biofilm functions against various bacterial strains. Carotenoid zeaxanthin inhibited virulence gene expression and biofilm formation of *P. aeruginosa via* quorum sensing inhibition (Gokalsin et al., 2017). Biofilm formation of Gram-positive strains, such as *S. aureus*, *Bacillus subtilis*, and *Listeria monocytogenes*, were also inhibited by carotenoids isolated from skull and exterior covering body parts of prawn (Jeyachandran et al., 2020). These reports lead us to speculate that Δ*dra0033* strain-derived carotenoids were involved in its residual effects of biofilm inhibition. Besides, we previously demonstrated that single mutant Δ*dra0033* strain produced 79.8% less DeinoPol than that of WT strain. However, it still expressed DeinoPol in a detectable amount, which may function, at least in part, as an inhibitor against *S. aureus* biofilm. To date, there have been few studies on the anti-biofilm substance of *D. radiodurans*, so it can only be speculated. Further studies are needed to determine the existence and mechanisms of *D. radiodurans*-produced biofilm regulating molecules other than DeinoPol.

Live beneficial bacteria, such as probiotics, generally used as foods or dietary supplements rather than drugs due to its risk and safety issues (Venugopalan et al., 2010). However, various bacterial products, such as exopolysaccharides, have been approved for medical applications in human since the mid-nineteenth century (Moscovici, 2015). For example, dextran or hyaluronic acid/hyaluronan produced by bacteria are now applicated in chronic wound healing, osteoarthritis treatment, or plasma volume expansion for controlling wounds shock (Necas et al., 2008; Nwodo et al., 2012). DeinoPol contents and structure have already been described in our previous report (Lin et al., 2020) and its anti-biofilm efficacy *in vitro* and *in vivo* was also demonstrated in this study. Thereafter, if clinical trials demonstrate acceptable effects of DeinoPol, it could be used clinically as a standalone antimicrobial agent or an additive to antibiotics to treat biofilm-associated infections.

Taken together, the results of this study suggest that DeinoPol is a major constituent of *D. radiodurans* that inhibits *S. aureus* biofilm formation. DeinoPol can be used as a potential alternative or additive dispersing agent for the treatment of infectious diseases caused by *S. aureus* biofilms.

## Data Availability Statement

The original contributions presented in the study are included in the article/Supplementary Material, further inquiries can be directed to the corresponding author/s.

## Ethics Statement

The animal study was reviewed and approved by the Institutional Animal Care and Use Committee of Korea Atomic Energy Research Institute.

## Author Contributions

KA and HS conceived the idea and contributed to the discussion of the results followed by writing and reviewing the manuscript. KA, FC, SH, and HS designed the experiments. FC, KA, JZ, and HJ performed the experiments, and/or interpreted the data. M-KK, KK, J-IC, SH, and SL provided critical comments and contributed to the discussion of the results followed by writing and reviewing the manuscript. All authors contributed to the article and approved the submitted version.

## Conflict of Interest

The authors declare that the research was conducted in the absence of any commercial or financial relationships that could be construed as a potential conflict of interest.

## Publisher’s Note

All claims expressed in this article are solely those of the authors and do not necessarily represent those of their affiliated organizations, or those of the publisher, the editors and the reviewers. Any product that may be evaluated in this article, or claim that may be made by its manufacturer, is not guaranteed or endorsed by the publisher.
