# Supplementary material for: Deinococcus radiodurans Exopolysaccharide Inhibits Staphylococcus aureus Biofilm Formation
Source: Front Microbiol. 2021 Dec 24;12:712086. doi: 10.3389/fmicb.2021.712086 (PMC8739996; doi:10.3389/fmicb.2021.712086)
Supplement: Supplementary file 1 [file Table_1.DOCX]

Supplementary Table 1. Bacterial strains used in this study

| *Deinococcus radiodurans* | | |
| --- | --- | --- |
| Strain | Relevant characteristics | Reference or source |
| R1 | ATCC13939 | Laboratory stock |
| DRA0033 | R1, Δ*dra0033* | (Lin et al., 2020) |
| KCTC13953BP | Isolates from Baekrokdam in Jeju island | Laboratory stock |
| KCTC13954BP | Isolates from Baekrokdam in Jeju island | Laboratory stock |
| KCTC13955BP | Isolates from Baekrokdam in Jeju island | Laboratory stock |
| *Staphylococcus aureus* | | |
| Strain | Relevant characteristics | Reference or source |
| RN4220 | Restriction-negative laboratory strain | Laboratory stock |
| USA300 LAC | MRSA clinical isolate | (Voyich et al., 2006) |
| MW2 | MRSA clinical isolate | (Baba et al., 2002) |
| Mu50 | MRSA clinical isolate | (Kuroda et al., 2001) |

Supplementary Table 2. List of primers used for qPCR

| Gene | Primer Sequences (5’ - 3’） |
| --- | --- |
| *icaA* (intercellular  adhesion A) | Forward: GGCTGTATTAAGCGAAGTCAGACA  Reverse: GCCAACGTCGACAACTGCAC |
| *icaD* (intercellular  adhesion D) | Forward: AGCCCAGACAGAGGGAATAC  Reverse: ATCCAAAAGACACAAGATATAGCGA |
| *icaB* (intercellular  adhesion B) | Forward: CAGGTCATGTTGGGGAAGAA  Reverse: TGCAAATCGTGGGTATGTGT |
| *icaC* (intercellular  adhesion C) | Forward: TCACGATACCGTGCTACACT  Reverse: ACCCATATATGCACCTAAGAAGAA |
| *fnbA* (fibronectin-binding protein A) | Forward: ATACAAACCCAGGTGGTGGT  Reverse: CTCACTGCGCCAGTTACAAT |
| *fnbB* (fibronectin-binding protein B) | Forward: CAAGGCGACGGCAAAGATAA  Reverse: ATTCATGCTTCTCCACTGGC |
| *clfA* (clumping factor A) | Forward: TGTCATGGGACAACGAAGTAGCA  Reverse: TCACCAGGCTCATCAGGTTGT |
| *clfB* (clumping factor B) | Forward: GGTGGTGGAAGTGCTGATGG  Reverse: TGGGTCTGGACTTGGTTCTGG |
| *Fib* (fibrinogen binding protein) | Forward: CAGCAGCGAAAACTGATGC  Reverse: TGTGCACTGACAGTATGTGTT |
| *ebps* (elastin-binding protein) | Forward: TCGACTGAGGATAAAGCGTCTCA  Reverse: CCAGCCAAACCTGCTGTTCC |
| *eno* (laminin binding  protein) | Forward: CAGTAGGTGACGAAGGTGGT  Reverse: TGTAACCAGCTGCTTCGATTG |
| *cna* (collagen binding  protein) | Forward: GGTTTGGGAAGGCACTCAAA  Reverse: TCTCTGCTTTGTCTACTGGTGT |
| *gyrB* (gyrase B) | Forward: CGCACGTACAGTGGTTGAAA  Reverse: ACGCTGATTTACGACGTGTT |

**References**

Baba, T., Takeuchi, F., Kuroda, M., Yuzawa, H., Aoki, K., Oguchi, A., et al. (2002). Genome and virulence determinants of high virulence community-acquired MRSA. *Lancet* 359(9320)**,** 1819-1827. doi: 10.1016/s0140-6736(02)08713-5.

Kuroda, M., Ohta, T., Uchiyama, I., Baba, T., Yuzawa, H., Kobayashi, I., et al. (2001). Whole genome sequencing of meticillin-resistant *Staphylococcus aureus*. *Lancet* 357(9264)**,** 1225-1240. doi: 10.1016/s0140-6736(00)04403-2.

Lin, S.M., Baek, C.Y., Jung, J.H., Kim, W.S., Song, H.Y., Lee, J.H., et al. (2020). Antioxidant Activities of an Exopolysaccharide (DeinoPol) Produced by the Extreme Radiation-Resistant Bacterium *Deinococcus radiodurans*. *Sci Rep* 10(1)**,** 55. doi: 10.1038/s41598-019-56141-3.

Voyich, J.M., Otto, M., Mathema, B., Braughton, K.R., Whitney, A.R., Welty, D., et al. (2006). Is Panton-Valentine leukocidin the major virulence determinant in community-associated methicillin-resistant *Staphylococcus aureus* disease? *J Infect Dis* 194(12)**,** 1761-1770. doi: 10.1086/509506.
